# Adenosinergic Pathway and Linked Suppression: Two Critical Suppressive Mechanisms of Human Donor Antigen Specific Regulatory T Cell Lines Expanded Post Transplant

**DOI:** 10.3389/fimmu.2022.849939

**Published:** 2022-03-17

**Authors:** Sudipta Tripathi, Paloma L. Martin-Moreno, George Kavalam, Brittany L. Schreiber, Ana Maria Waaga-Gasser, Anil Chandraker

**Affiliations:** ^1^ Renal Division, Brigham and Women’s Hospital, Harvard Medical School, Boston, MA, United States; ^2^ Nephrology Department, Clinica Universidad de Navarra, Instituto de Investigación Sanitaria de Navarra, Pamplona, Spain

**Keywords:** kidney transplantation, allo-antigen specific Tregs, cell therapy, adenosinergic pathway, linked suppression

## Abstract

Regulatory T cells are an important component of an immune response shaping the overall behavior to potential antigens including alloantigens. Multiple mechanisms have been shown to contribute towards developing and sustaining a immunological regulatory response. One of the described contact dependent suppressive mechanisms regulatory cells have been shown to utilize is through the production of adenosine from extracellular ATP mediated by CD39 and CD73. In this study we demonstrate that the adenosinergic pathway plays a major role in the suppressive/regulatory effects antigen specific regulatory T cell enriched lines (ASTRLs) that have been of expanded *ex vivo* from stable kidney transplant patients. We have previously shown that these ASTRL cells are capable of suppressing alloimmune responses *in vitro* and significantly prolonging allograft survival in an animal model of kidney transplantation. For this study nineteen ASTRLs were expanded from 17 kidney transplant patients by repeated stimulation of recipient peripheral blood mononuclear cells with donor specific HLA-DR peptides. All 19 ASTRLs showed upregulation of numerous markers associated with regulatory cells and were able to inhibit donor antigen specific T cell proliferation in a dose dependent fashion. ASTRLs suppressed indirect and direct alloimmune responses compatible with our previous animal study findings. Upregulation of both CD39 and CD73 was observed post expansion and ASTRLs demonstrated extracellular hydrolysis of ATP, indicating functionality of the upregulated proteins. We also showed that inhibition of the adenosinergic pathway using inhibitors of CD39 resulted in abrogation of suppression and increased antigen specific T cell proliferation. This demonstrates that the main mechanism of action of the suppressive activity donor peptide driven ASTRLs generated from kidney transplant patients is the adenosinergic pathway. Furthermore this suggests the possibility that combining infusion of Tregs with other treatments, such as adenosine receptor agonists or increasing CD39 expression in the grafts may further enhance a regulatory response to the allograft and possibly achieve transplantation tolerance.

## Introduction

Kidney transplantation is the preferred treatment for patients with end stage kidney disease. Despite significant improvements in short term allograft survival, long-term graft survival has not changed dramatically in many years, related primarily to an ongoing chronic immune response directed against the allograft and the associated toxicities of maintenance immunosuppressive medications. While maintenance immunosuppression is important in blunting the immune response to the allograft, it has also been demonstrated that stable allograft function is partially dependent on an immunoregulatory response to the allograft (1). In animal models of transplantation it is possible to constrain an effector alloimmune response by increasing the immunoregulatory response towards the allograft in either the absence or combination with very low doses of standard immunosuppression (2).

While induction of immune tolerance has traditionally been seen as the best path towards avoiding the negative effects of conventional maintenance immunosuppression for transplant recipients, an integral part of most of these strategies is the need for an induction phase utilizing donor tissue, making them impractical for translation into a deceased donor setting. An alternative approach that overcomes the need to have access to donor tissue, would be to utilize prefabricated donor peptides to expand a regulatory T cell population that demonstrate linked or bystander suppression, where a regulatory cell is able to suppress an immune response to a variety of associated donor antigens, even when derived using a single antigen.

Regulatory T cells (Tregs) are an important component of the alloimmune response, acting as ‘professional’ suppressors of an aggressive immune response, their importance in maintenance of allograft function has been shown in multiple *in vitro* and *in vivo* models (3, 4). Our previous work both in rats and humans has also shown that an immuno regulatory response towards the allograft exists and contributes towards long term acceptance and survival of stable allografts (2, 5). In a rat model of kidney transplantation, it has been demonstrated that ’Th2 clones’, derived from previously transplanted animals with stable allograft function specifically regulated the activation of Th1 clones (derived from rejecting transplanted animals), maintained graft function and prevented chronic rejection of the allograft (2, 5). In humans, we have shown that higher percentages of circulating regulatory T cells were found in transplanted patients with stable graft function irrespective of their maintenance immunosuppressive regimen. Patients with unstable allograft function showed a more inflammatory response towards donor antigen *in vitro*. These studies clearly demonstrated the role of donor antigen specific Tregs in allograft acceptance and chronic rejection. We have also shown that it is possible to expand donor antigen specific regulatory T cell enriched lines (ASTRLs) from kidney transplant patients with stable kidney function using manufactured peptides corresponding to mismatched donor HLA DR antigens. As expected, these ASTRLs had increased percentages of CD4^+^ CD25^+^ T cells that showed Treg associated gene expression profiles (5).

The potential therapeutic use of Tregs as regulators of the immune response to the allograft has led to an interest in the development of different protocols to expand Tregs *ex vivo* through either antigen specific or non-specific methods. In antigen-specific expansion, Tregs are typically exposed to alloantigen through the ‘direct pathway’ of alloantigen presentation by utilizing donor cells such as B cells and dendritic cells (6, 7), or an ’indirect pathway’ of presentation through stimulation by autologous antigen presenting cells along with donor peptide (8). Donor alloantigen specific Tregs have been shown to be five to ten times more effective than non-specific polyclonal Tregs (9, 10). Many of these Treg directed therapies are currently in clinical trials for various autoimmune diseases and solid organ transplantation (11, 12).

The current study is an extension of our prior work showing that a donor alloantigen specific regulatory response exists in conventionally immunosuppressed patients and contributes significantly towards long-term allograft acceptance and that furthermore it is possible to expand an immunoregulatory cell population from the peripheral blood mononuclear cells (PBMCs) of stable kidney transplant recipients utilizing donor derived HLA-DR antigens. We also explore the suppressive phenotype and function of these *ex vivo* expanded cells and show that the suppressive effect of these cell lines is mediated through the adenosinergic pathway and that single antigen driven ASTRLs demonstrate linked suppression towards other donor antigens.

## Materials and Methods

### Study Subjects

A total of 45 kidney transplant recipients (KTRs) with one or more DR mismatches (DR1, DR4, DR15, and DR 17) with the donor were included in the study. The local institutional ethics committee approved the study protocol and all patients gave written informed consent.

Patients were treated with double or triple immunosuppressive therapy including tacrolimus, except in three cases that received everolimus or belatacept instead of tacrolimus.

Blood samples were obtained at various post-transplant visits and PBMCs were isolated and expanded *ex vivo* from 17 of the 45 patients based on available peptides for recipients DR mismatch.

These *ex vivo* expanded donor Antigen Specific T cell enriched immuno-Regulatory cell Lines are referred to as ASTRLs in the rest of this paper for ease and simplicity of description.

### HLA-DR Donor Specific Peptides

A panel of peptides was synthesized corresponding to the full-length β-chain hypervariable regions of HLA-DRB1* 0101, 1501, 0301 and 0401 (ProImmune, Littlemore, UK), as previously reported (1).

### HLA-DR-Specific ASTRLs

Peripheral blood samples were collected from KTRs at various visits post transplantation and PBMCs were isolated by density gradient centrifugation method using Lymphoprep (Stemcell). The cells were then expanded *ex vivo* or frozen in LN2 for future use. Briefly, 10 × 10^6^ PBMCs were cultured in Immunocult serum free culture medium (Stemcell), containing 100 U/ml penicillin, 100 μg/ml streptomycin, 100 μg/ml L-glutamine, 5 mmol/l HEPES, 1% nonessential amino acids, and 1 mmol/l sodium pyruvate (Gibco), and 2-mercaptoethanol. These ‘responder’ PBMCs were repeatedly stimulated at 7–10-day intervals with autologous irradiated (10–15 Gy) ‘stimulator’ PBMCs acting as antigen-presenting cells loaded with the mismatched donor-derived HLA-DR allopeptides (50 μg/ml) in the presence of IL-2 (100 IU/ml) as described previously (1). All ASTRLs were cultured at 37°C in a humidified 5% CO_2_ incubator and harvested after four to five cycles of stimulation.

### Flow Cytometric Analysis

ASTRLs and PBMCs were immunophenotyped for various cell surface markers by flow cytometry with fluorophore conjugated human anti-CD3, anti-CD4, anti-CD8, anti-CD25, anti-CD127, anti-CD39 and anti-CD73 anti-CTLA4, anti-GITR, anti-ICOS, anti-CD45RA, anti-CD226, anti-LAP, anti-GARP, anti-CD56, anti-CD16, anti-CD19, anti-CD11b, anti-CD38, anti-CD27, and anti-CD24 (Biolegend). The data were acquired using a Canto II cytometer (BD Biosciences) and analyzed using Flowjo. The gating strategy for phenotyping included initial gating of a live PBMC population followed by the CD3^+^CD4^+^ population. The expression of CD25, CD127, CD39, and CD73 were expressed as % of the CD3^+^CD4^+^ population.

T cell proliferation and suppression was determined by CFSE dye dilution of the responder cells. Analysis of CFSE distribution was performed on Flowjo Proliferation platform and data are represented by Replication Index (RI). RI, determines the fold-expansion of only the responding cells, and it is the average number of divisions that all cells have undergone after they had been stained by a cell proliferation dye (13). The percentage of suppression was calculated from proliferation and suppression values.

### Proliferation and Suppression Assays

In order to test the ability of ASTRLs to regulate an immune response against donor antigen, proliferation assays were set up. For the indirect pathway of allostimulation 1 × 10^6^ CFSE stained PBMCs were used as responders and stimulated with donor-mismatched HLA-DR allopeptide and autologous irradiated PBMCs (2 × 10^6^) for 72 h in a humidified 5% CO_2_ incubator in a 96 well U bottom plate. For the direct allorecognition proliferation assays, irradiated whole *donor* PBMCs were used as stimulators. In other experiments CD3/CD28 beads were also used to induce non-specific T cell proliferation. Proliferation was assessed by dilution of CFSE. Stimulated PBMCs were cultured in presence or absence of ASTRLs at a ASTRL: PBMC ratio ranging from 1:2 to 1:16, or in some cases 1:256, in the suppression assays. In some experiments a transwell plate was used instead of a 96 well u bottom plate. Other experiments included the addition of CD39 inhibitor (POM1, 10 μg/ml), to the suppression assay. All assays were set up in triplicates and performed multiple times (n = 5).

### Cytokine and Chemokine Measurement

Cytokines and chemokines were measured in the culture supernatants from suppression assays by Luminex using multiplex assay kits from Thermofisher following the protocol of the manufacturer.

### ATP Hydrolysis Assay

Extracellular ATP hydrolysis and production of adenosine by ASTRLs was determined by measuring the production of inorganic phosphate, a byproduct of the same reaction, using malachite green colorimetric assay kit from Anaspec according to the protocol of the manufacturer (14).

### Statistical Analysis

Results are expressed as mean ± s.d. Characteristics of patients, phenotype, and functional data were compared by the Student’s t-test or ANOVA as appropriate. Each experimental condition was repeated three times. A p <0.05 was considered significant.

## Results

### Demographic and Clinical Characteristics of KTRs

The study included samples taken from a total of 45 KTRs with one or more donor DR mismatches. Nineteen individual ASTRLs were expanded *ex vivo* from the PBMCs of 17 subjects. The demographic data of these 17 subjects are presented in **Table 1**.

**Table 1:** Demographic data of the 17 patients from whom ASTRLs were expanded.

	n = 17
Age (years)	51.6 ± (16)
Sex	
Female (%)	47
Male (%)	53
HLA mismatch (number)	4.5 ± (1.3)
Serum creatinine (mg/dl)	1.5 ± (0.4)
Proteinuria >0.5 g/24 h	2
Acute/chronic rejection (total number)	4/1
Type of donor	
Deceased (%)	47
Living (%)	53
Time (months) between date of Transplant and C1	17 ± (21.5)

HLA, human lymphocyte antigen; C1, the first blood collection.

Values for age, HLA mismatch, serum creatinine, and time between date of Transplant and C1 are expressed as mean ± (s.d.).

In total, 14 of the patients received thymoglobulin induction therapy. With respect to maintenance immunosuppressive treatment, 14 patients received tacrolimus, 10 of whom received in combination with mycophenolate mofetil (MMF) or mycophenolic acid (MPA) and steroids, 1 patient received everolimus with MMF, and 2 patients received belatacept and steroids, of which one was also treated with MMF and the other with azathioprine.

All patients had stable renal function, however four of the patients had had a prior episode of treated acute rejection.

### *Ex Vivo* Expanded ASTRLs Express a Regulatory Phenotype

**Figure 1:**
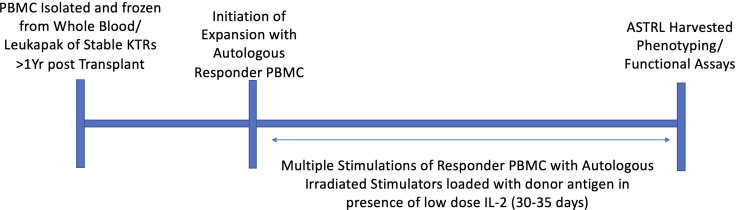
Graphical representation of *ex vivo* expansion of ASTRL. ASTRLs were expanded from autologous PBMC of stable kidney transplant patients through multiple stimulations in presence of donor antigen and low dose IL-2 using the indirect pathway of allorecognition. A typical expansion process takes about 30 to 35 days. At the end of the expansion cells were harvested and phenotypic and functional characterization of the ASTRLs are performed.

**Figure 2:**
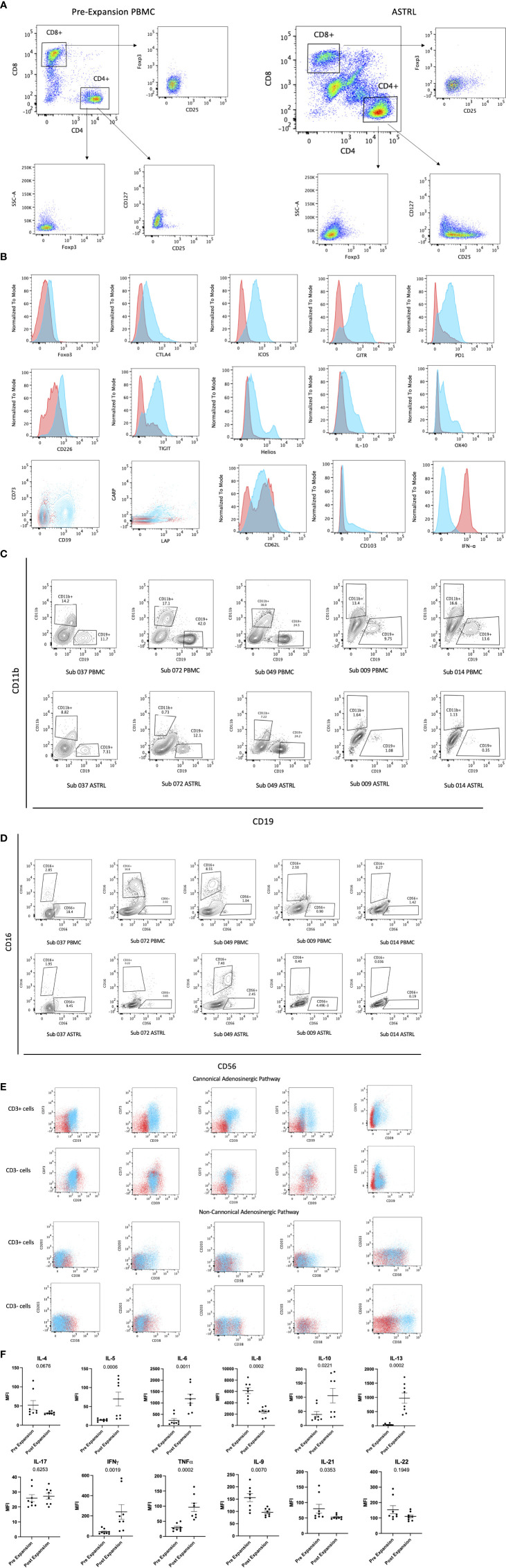
**(A)** Phenotypic characterization of ASTRL. Flow cytometry plots show expression of CD25, CD127, and Foxp3 in CD4^+^ and CD8^+^ T cells of pre expansion PBMC and ASTRL. **(B)** Expression of various conventional T regulatory cell markers on CD4^+^ T cell subsets of pre-expansion PBMC and ASTR L. Histograms and contour plot overlays show PBMC in red and ASTRL in blue. **(C)** ASTRLs constitute a heterogenous cell population. Figures show a comparison of CD19^+^ B cell and CD11b^+^ myeoid cell populations in pre-expansion PBMC (top panel) and ASTRL (bottom panel) from five different subjects. **(D)** ASTRLs constitute a heterogenous cell population. NK cell population in ASTRLs (bottom panel) and pre expansion PBMC (top panel) are shown from five different subjects. **(E)** ASTRLs upregulate the surface expression of the molecules belonging to canonical (CD39 and CD73) and non-canonical (CD38 and CD203) adenosinergic pathways. Figures on the top panels show the expression of CD39 and CD73 on the CD3^+^ and CD3^−^ cell populations of ASTRL in blue and pre-expansion PBMC in red. The bottom panel shows the expression of CD38 and CD203 in CD3^+^ and CD3^−^ subsets of ASTRL in blue and pre-expansion PBMC in red. **(F)** Differentially expressed cytokines in the expansion media of ASTRLs. Post expansion, on days 30–35 ASTRLs were harvested and production of various cytokines in the spent expansion media were measured by Luminex.

ASTRLs were expanded *ex vivo* from the PBMCs of KTRs by multiple stimulations using autologous irradiated APCs loaded with donor specific HLA-DR allopeptides. The donor specific allopeptides were chosen based on the tissue typing data obtained prior to transplantation. **Figure 1** shows a typical ASTRL expansion process. The entire process takes between 30 and 35 days at the end of which ASTRLs are harvested. Post expansion, each ASTRL was analyzed by flow cytometry to determine the phenotypic diversity and distinction between the ASTRL and the initial pre-expansion PBMC population. We observed an increase in the percentage of CD4^+^ T cells in the ASTRL. The ASTRL CD4^+^ T cells were CD25^hi^ and CD127^lo^ and expressed a small increase in Foxp3 expression (**Figures 2A, B** **)**. As the ASTRLs were enriched in CD4^+^ T cells, we next determined the expression of various conventional Treg markers on the CD4^+^ T cell subset of both post-expansion ASTRL and pre-expansion PBMC. Multiple Treg surface markers that were significantly upregulated in the ASTRLs include CD39, CD73, cytotoxic T-lymphocyte-associated protein 4 (CTLA4), Glucocorticoid-induced tumor necrosis factor receptor-related protein (GITR), inducible co-stimulator (ICOS), Latency-associated peptide (LAP) and Glycoprotein A repetitions predominant (GARP), T-cell immunoreceptor with Ig and ITIM domains (TIGIT), Helios, TNF receptor super family 4 (OX40/TNFRSF4), CD103 and IL-10 (**Figure 2B**).

We observed that the percentage of enriched T cells in the ASTRLs varied widely between the various subjects and the range of CD3^+^CD4^+^ T cells was 20–50%. ASTRLs showed an increased expression of CD3, CD4, and CD25 in comparison to the pre expanded PBMC population. No significant change in the expression of CD127 was observed (**Figure 2A**). An increase in Foxp3 expression was also observed (**Figure 2B**). The ASTRLs contained a CD3^−^ non-T population comprising mostly of natural killer cells. We also determined the percentage of CD19^+^ B cell and CD11b^+^ monocyte populations and these populations were consistently lower in the ASTRLs in comparison to that of the pre-expansion PBMC (**Figures 2C, D** **)**. However, the percentage of monocytes, B and NK cell populations showed wide variations between subjects.

We consistently observed increased expression of the surface markers associated with the adenosinergic pathway in the ASTRLs. Both the CD3^+^ and CD3^−^ cell populations of ASTRLs upregulated the canonical (CD39 and CD73) and the non-canonical (CD38 and CD203) phenotypic markers (**Figure 2E**). Again, there was a wide variation (20–60%) in the percentage of cells expressing CD4^+^CD39^+^ and CD4^+^CD73^+^, between the various ASTRLs.

It is noteworthy that unlike in murine cells CD39 and CD73 were not co-expressed on the same CD4^+^ T cell population (15). However, in most of the ASTRLs CD39 and CD73 are expressed on distinct populations of CD4^+^ T cells and coexpressed on a smaller subset (**Figure 2B**). The same is true for CD38 and CD203 expression.

**Figure 2F** shows the differential production of various cytokines in the culture supernatant of the pre-expansion PBMC and resting post stimulation ASTRLs. IL-10 and IL-13 production was significantly increased, IL-8 production significantly decreased, and IL-17 showed no change.

### ASTRLs Suppress Donor Antigen Specific T Cell Proliferation

**Figure 3:**
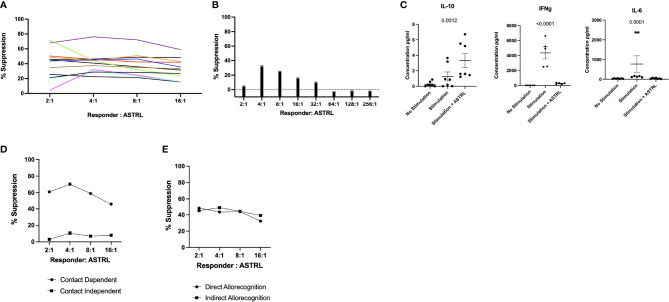
Functional Characterization of ASTRLS. ASTRLs suppress donor antigen specific proliferation of T effector cells. **(A)** ASTRLs expanded from various KTRs suppress donor antigen specific proliferation of T effector cells. **(B)** The suppressive effect of ASTRLs on donor antigen specific proliferation increasing ASTRL : Responder ratio. **(C)** Cytokine production in the culture supernatant of suppression assay by Luminex. **(D)** Suppressive ability of ASTRL in contact dependent and contact independent transwell assay. **(E)** Suppressive effect of ASTRL to responder cell proliferation in response to a direct allorecognition using donor cells as stimulators and an indirect allorecognition using autologous irradiated stimulators loaded with donor antigens.

The functional characterization of the ASTRLs was determined next by assessing their immunosuppressive function to inhibit antigen specific cell proliferation in a standard suppression assay. We observed that the ASTRLs were able to inhibit donor alloantigen specific T cell proliferation. **Figure 3A** shows the percentage of suppression by ASTRLs expanded from multiple KTRs. All the ASTRLs show maximum suppressive capacity at a ratio of 1 ASTRL:4 Responders. As would be expected the suppressive effect of ASTRLs decreased when the ratio of ASTRL cells to responders was increased. **Figure 3B** shows the dilution of the suppressive capacity of a single ASTRL that shows the highest % suppression at ASTRL : Responder ratio of 1:4 and no suppression at ASTRL : Responder ratio of 1:64. The addition of ASTRLs also significantly decreased production of IFN-γ and IL-6 and increased production of IL-10 in the assay culture supernatant (**Figure 3C**) in the proliferation assay.

To further understand the mechanism of suppression of the ASTRLs a classical transwell system of suppression assay was used to determine if the suppressive effect of ASTRLs is dependent on cell–cell contact. We observed that the ASTRLs lost their ability to suppress donor antigen specific T cell proliferation when separated by a semi permeable membrane demonstrating that the ASTRL mediated suppression is dependent on cell–cell contact. **Figure 3D** shows that the suppressive effect of ASTRLs is contact dependent and in the presence of a barrier in the form of a transwell insert, ASTRLs are unable to suppress antigen specific proliferation of responder cells.

Allorecognition pathways play an important role in alloantibody production and chronic rejection in transplantation. We next determined the efficacy of ASTRLs in suppressing the effector response to the same donor antigen presented either by the direct allorecognition pathway using donor cells as stimulators or by the indirect pathway using autologous stimulators loaded with allopeptides. ASTRLs effectively suppressed proliferation of responders stimulated by both direct and indirect allorecognition as shown in **Figure 3E**. These results demonstrate that ASTRLs efficiently suppress donor antigen specific effector response *in vitro* in a contact dependent manner irrespective of the allorecognition pathway.

### ASTRLs’ Suppressive Ability is Mediated Through the Adenosinergic Pathway

Based on our previous observations that ASTRLs upregulate the expression of two important ectoenzymes CD39 and CD73 of the canonical adenosinergic pathway we next determined the contribution of this pathway to the suppressive capacity of the ASTRLs. The adenosinergic pathway works by hydrolyzing extracellular ATP released during inflammation to produce adenosine, an immunosuppressive molecule. Human regulatory T cells that produce higher levels of adenosine from ATP are known to be more suppressive.

**Figure 4:**
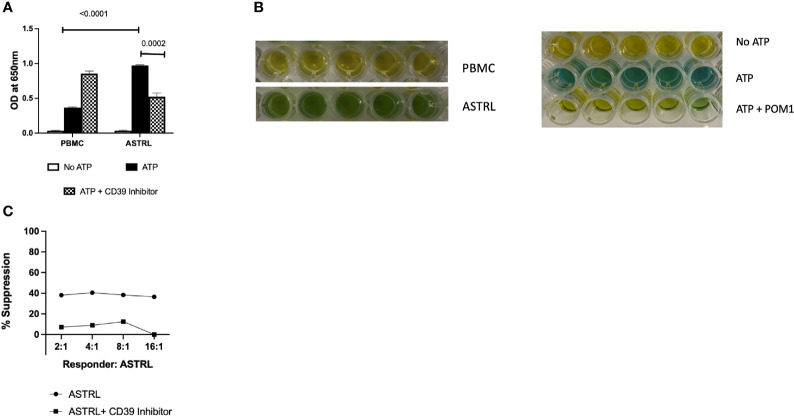
ASTRL hydrolyze extracellular ATP in a CD39 dependent manner. **(A)** ASTRLs Extracellular ATP (eATP) hydrolysis by ASTRL and in presence or absence of POM1, a CD39 inhibitor. **(B)** Malachite Green assay, that detects the presence of inorganic phosphate (green color) resulting from ATP hydrolysis, showing eATP hydrolysis by ASTRL and PBMC. **(C)** Effect of CD39 inhibition (presence of POM1) on suppressive effect of ASTRL on antigen specific proliferation of responder cells.

The ability of ASTRLs to hydrolyze extracellular ATP was determined using Malachite Green Assay. **Figures 4A, B** show that ASTRLs were able to hydrolyze ATP at a significantly higher rate in comparison to the pre-expansion PBMC and ATP hydrolysis by ASTRLs is inhibited in presence of a CD39 inhibitor, POM1. We further confirmed the contribution of the adenosinergic pathway to the suppressive effect of ASTRLs in a suppression assay in the presence or absence of POM1. The use of POM1 resulted in the abrogation of suppression and increase in antigen specific T cell proliferation (**Figure 4C**). It is evident from these experiments that the regulatory function of ASTRLs is mediated through the adenosinergic pathway *via* upregulation of CD39 and CD73 leading to the generation of the immunosuppressive molecule, adenosine.

### ASTRLs Exhibit Bystander Suppression to Other Donor Antigens

**Figure 5:**
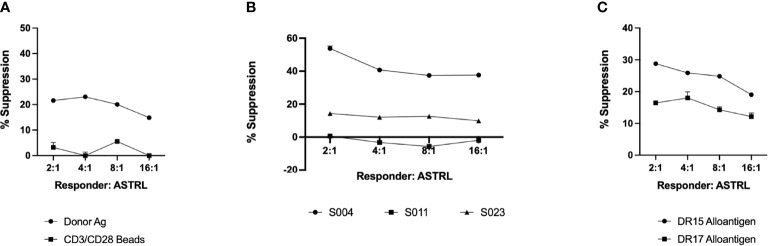
ASTRLs exhibit antigen specific, individualized/patient specific and bystander suppressive function. **(A)** Suppressive effect of ASTRL on non-specific proliferation of responder cells using CD3/CD28 beads. **(B)** Suppressive effect of ASTRL on antigen specific proliferation of autologous and heterologous responders that share the same donor antigen. **(C)** Bystander suppressive effect of ASTRL on antigen specific proliferation of autologous responders to multiple donor antigens.

Our next aim was to determine if ASTRLs suppress the effector response of additional donor antigens. This phenomenon, called bystander suppression, is a property of Tregs and is also referred to as linked suppression. Our data mentioned above showed the ability of ASTRLs to suppress the effector response to direct allorecognition (**Figure 3E**). This indicates that ASTRLs are able to suppress the effector response to multiple donor antigens, irrespective of their own antigen specificity. To further characterize and understand the bystander suppressive effects of ASTRLs we focused on subjects that had two donor DR mismatches. **Figure 5C** shows that ASTRLs expanded with one donor antigen (in this case DR15) specificity successfully suppress the T effector response to the other mismatched donor antigen (DR17) thus demonstrating linked/bystander suppression. To our knowledge this is the first report of an *ex vivo* expanded regulatory T cell enriched population demonstrating bystander suppression *in vitro* to other donor antigens. This interesting result directed us to the next obvious question of subject/patient specificity of ASTRLs. To ascertain that ASTRLs regulatory activity was donor specific we selected groups of subjects that had only one DR mismatch and shared the same DR mismatch with their respective donors. A single ASTRL was expanded from one subject (S004) against the donor DR (DR4) and used in suppression assays where PBMCs from three different responders were stimulated with their respective autologous APCs loaded with the same DR4 in presence of the S004 ASTRL. **Figure 5B** shows that ASTRL expanded from S004 was able to suppress the proliferation of the autologous responders only. There was no suppression of responder cell proliferation against the same DR of one of the two subjects by the ASTRL and significantly reduced suppression against the other. In addition, ASTRLs did not show any significant suppression of effector response to nonspecific stimulation (**Figure 5A**).

## Discussion

Specific immunoregulation and/or tolerance towards an allograft without the need for immunosuppression allows the immune system to appropriately respond towards infections and malignancies while maintaining graft function without the associated side effects of immunosuppressive medications. The current regulatory T cell-based immunotherapies targeted towards solid organ transplantations strive to achieve this ideal immunoregulatory state. Here we report the feasibility of expanding a regulatory T cell-based therapy, ASTRLs, from the PBMCs of KTRs post transplantation. Our study focuses on ASTRL expansion, phenotypic, and functional characterization from multiple kidney transplant recipients. We were able to show that we can generate a regulatory cell population from all patients through a simple robust *ex vivo* expansion method. In addition, our data show that the ASTRLs express a regulatory phenotype, are immunosuppressive in function and the suppressive activity is mediated through the adenosinergic pathway.

Even as the alloimmune response creates an aggressive effector response against ‘foreign’ antigens it also simultaneously creates a regulatory T cell response comprised of phenotypically and functionally dynamic and diverse subpopulations. However, most of the current regulatory T cell-based therapies, focus on homogenous regulatory T cell populations that are phenotypically defined as CD4^+^ CD25^+^ CD127^−^ Foxp3^+^ (16, 17). Although multiple additional markers of human Tregs have since been identified and several recent studies suggest a phenotypically and functionally diverse human Treg population, none of these are taken into consideration in the emerging adoptive Treg based therapies (18). Our approach successfully expands a donor antigen specific heterogenous cell population enriched in CD4^+^ T cells that express various established regulatory markers.

It is important to consider here that the CD4^+^ T cell subset within an ASTRL is not the conventionally defined CD4^+^ CD25^+^ CD127^−^ Foxp3^+^ Treg population. Phenotypically the CD4^+^ T cell subset within the ASTRLs is heterogeneous and regulatory in nature. While within the ASTRLs CD25 expression is high and CD127 is low and Foxp3 is present, it is also noteworthy that there is an increased expression of many other molecules, like CTLA4, GITR, ICOS, TIGIT, GARP, LAP, OX40, Helios, PD1, IL-10, that are regulatory in nature on the total CD4^+^ subset and not only in the conventional Tregs. ASTRLs also overexpress the functional Treg marker CD39. CD39, the not so new check point inhibitor/mediator, is crucial in the maintenance of immune homeostasis. The balance between extracellular ATP and adenosine is controlled by the canonical adenosinergic pathway where CD39 enzymatic activity is the rate limiting step. CD39 is expressed by a variety of immune cells in addition to Tregs. Its function is primarily generating an immunosuppressive environment by degrading extracellular ATP to produce adenosine and neutralizing a proinflammatory environment. The ectoenzyme CD39 is expressed on a highly suppressive memory-like subset of Tregs (19), but recent reports using mass cytometry reveal that more than one subpopulation of human Tregs express CD39 and some of these subsets co-express CD73 (18). We observe very similar CD4^+^ T cell sub populations within ASTRLs that co-express CD39 and CD73 and/or independently express CD39 and CD73. CD39 has also been shown to be a better marker of Treg stability (20).

ASTRLs also contain a smaller subset of CD3^−^ cells that are heterogeneous and comprise of CD56^+^ NK cells, CD19^+^ B cells, CD11b + myeloid cells. The CD3^−^ subset also show surface expression of CD39, CD73, CD38, and CD203. CD39 expression on immune cells other than T cells are known to contribute towards an immunosuppressive microenvironment (21). High level expression of CD39 on CD8^+^ T cells and B cells are reported to be functionally immunosuppressive. The adenosinergic pathway is the primary mechanisms that mediate the suppressive ability of ASTRLs. The expression of CD39, CD73, CD38, and CD203 on the CD3^−^ population supports our approach of a heterogeneous immunoregulatory population that creates a bias towards a regulatory microenvironment.

Inflammation impedes transplantation tolerance and contributes to the development of chronic allograft rejection while activation of the adenosinergic pathway in response to ischemia/reperfusion injury is also shown to prolong allograft survival in various solid organ transplantations (22). Considering the critical role of CD39 in preventing inflammation, its established immunosuppressive role in tumor microenvironment and expression on highly suppressive Treg subsets it is imperative that the CD39 expression on ASTRLs are functional. Our data shows that CD39 expressed ASTRLs are functionally capable of hydrolyzing extracellular ATP and also suppressing donor antigen specific T cell proliferation and in the presence of a CD39 inhibitor lose their suppressive ability. It is important to consider here that CD39, combined with the other molecules of the adenosinergic pathway, plays an important role in rendering the ASTRLs immunosuppressive. It is also pertinent that as CD39 is expressed on a variety of immune cells, in a heterogeneous population like ASTRLs it may play a significant role in inducing an immunosuppressive microenvironment through the interaction between various cell types similar to a tumor microenvironment.

The anti-inflammatory properties of ASTRLs are also evident from our results showing increase in IL-13 and IL-10 and decrease in IL-8 production in the ASTRL expansion media and a significant reduction in IL-6 production by T cells in response to allostimulation in presence of ASTRL. Liu et al., describe that IL-13 production by the human Tregs is critical in the reduction of local proinflammatory response in lung injury specifically by controlling levels of IL-6 (23). Tregs producing IL-13 are Th2 biased, produce IL-10 and suppress CD4^+^ T cell proliferation (24), very similar to ASTRLs as described in both our previous (1) and current studies.

The work by Todo et al., showed that a heterogeneous Treg cell enriched product infused into a group of liver transplant patients was safe and effective in achieving immunosuppressive drug minimization and operational tolerance (25). However, there are some distinct differences between the expansion process of ASTRLs and the above mentioned Treg cell enriched product. As with nearly all of the other approaches within the literature the need for donor cells limits the use of approach of the group of Todo to only patients with living donors. Our approach, using autologous APCs loaded with donor antigen as stimulators, bypasses the need for donor cells in the expansion process broadening the clinical application to a larger population of organ transplant recipients. Our data shows that ASTRLs were successfully generated any post-transplant patient and 47% of the KTRs we expanded had received an organ from a deceased donor. The expansion of ASTRLs using the indirect pathway of allorecognition also make these cells more potent (26, 27). Efficacy of ASTRLs as an immunoregulatory population is even more compelling as demonstrated by the bystander suppression of other donor antigens. Bystander suppression enhances an immunosuppressive response creating immunoregulatory and/or tolerogenic microenvironment (28). To our knowledge this is the first-time evidence of the bystander suppression being demonstrated by a regulatory cell therapy product *in vitro*. We also show that this bystander suppression demonstrated by ASTRL is specific to the donor antigens and is individualized. The presence of bystander may not be that surprising as the PBMCs we collect were from non-rejecting transplanted patients who have been ‘primed’ to all donor antigens through the transplant process.

The extensive phenotypic characterization and identification of the mechanism of action of ASTRLs provides a well-defined if not comprehensive population of regulatory cells that successfully inhibit donor specific effector responses. It is prudent to discuss here that ASTRLs are a heterogeneous product, that contains B, NK, and CD8^+^ T cells, which are considered a potential concern in the cell therapy domain. However regulatory subsets of all of these cell types have been described in the literature and the expression of CD39 on CD3^−^ and CD8^+^ cells indicates that these cells are actually contributing to the regulatory environment. We would like to draw attention again here to the work of Todo et al., where a similar heterogeneous product has been shown to be safe and efficacious when infused into liver transplant patients (25, 29, 30). They have also shown that post expansion sorting adversely affects the suppressive function of these cells (29), akin to our observations (unpublished data). Our data show that the predominant mechanism of suppression in ASTRLs is mediated through the adenosinergic pathway and together with the T cells the non-T cells in the ASTRLs also upregulate the molecules of this pathway and contribute to the production of the immunosuppressive molecule adenosine.

In conclusion, we rationalize that ASTRLs, as a heterogeneous population, will likely create a bias towards a more robust regulatory microenvironment that results from the interaction of different subsets of immune cells rather than a small percentage of homogenous polyclonal Tregs.

## Data Availability Statement

The original contributions presented in the study are included in the article/supplementary material. Further inquiries can be directed to the corresponding author.

## Ethics Statement

The studies involving human participants were reviewed and approved by the Mass General Brigham Institutional Review Boards. The patients/participants provided their written informed consent to participate in this study.

## Author Contributions

ST and PLM performed majority of the experiments, analyzed the data and wrote the manuscript. GK and BLS performed some of the experiments. AW provided valuable insights and contributed to experimental and study design. AC contributed to study design, critically evaluated the data and helped shape the manuscript. All authors listed have made a substantial, direct, and intellectual contribution to the work and approved it for publication.

## Funding

The authors acknowledge the support of the Saxena Kidney and Pancreas Transplantation Research Fund in aiding the research efforts.

## Conflict of Interest

The authors declare that the research was conducted in the absence of any commercial or financial relationships that could be construed as a potential conflict of interest.

## Publisher’s Note

All claims expressed in this article are solely those of the authors and do not necessarily represent those of their affiliated organizations, or those of the publisher, the editors and the reviewers. Any product that may be evaluated in this article, or claim that may be made by its manufacturer, is not guaranteed or endorsed by the publisher.
